# Influence of Sulcus-Deepening Trochleoplasty on Patellofemoral Cartilage Integrity in Patients With Severe Trochlear Dysplasia at Short-term to Midterm Follow-up: A Case-Control Study

**DOI:** 10.1177/23259671251326052

**Published:** 2025-04-02

**Authors:** Jakob Ackermann, Georg C. Feuerriegel, Lazaros Vlachopoulos, Sandro F. Fucentese

**Affiliations:** † Department of Orthopaedics, Balgrist University Hospital, University of Zurich, Zurich, Switzerland; ‡ Department of Radiology, Balgrist University Hospital, University of Zurich, Zurich, Switzerland; Investigation performed at Balgrist University Hospital, University of Zurich, Zurich, Switzerland

**Keywords:** patellofemoral instability, trochlear dysplasia, trochleoplasty, osteoarthritis, cartilage

## Abstract

**Background:**

Sulcus-deepening trochleoplasty is a well-established treatment option for patients presenting with severe trochlear dysplasia and patellar instability. However, concerns remain regarding its influence on cartilage integrity in the patellofemoral (PF) joint.

**Purpose:**

To assess the midterm effect of trochleoplasty on PF cartilage integrity in patients with severe trochlear dysplasia treated for patellar instability.

**Study Design:**

Cohort study; Level of evidence, 3.

**Methods:**

A total of 75 patients with high-grade trochlear dysplasia (Dejour types B and C) who underwent patellar stabilizing surgery for patellar instability at a single institution were included. Of these, 42 patients underwent patellar stabilizing surgery without trochleoplasty (group I), while 33 patients underwent thin-flap sulcus-deepening trochleoplasty as part of their surgical treatment (group II). Preoperative and postoperative magnetic resonance imaging scans were retrospectively assessed to evaluate PF cartilage, grading from 0 (intact) to 4 (full-thickness lesion) for the medial, central, and lateral patella as well as the medial, central, and lateral trochlea. Associations between patient-specific characteristics, anatomic parameters, and chondral integrity were also assessed.

**Results:**

Patients underwent patellar stabilizing surgery at a mean age of 23.2 ± 8.0 years with a body mass index of 25.5 ± 5.0 kg/m^2^. Postoperative magnetic resonance imaging was performed at a mean of 35.2 ± 26.3 months (range, 6-118 months). Patients in group II were slightly older (25.0 ± 7.5 vs 21.8 ± 8.2 years, respectively; *P* = .032) and had a significantly higher preoperative tibial tubercle–trochlear groove distance (18.4 ± 4.0 vs 14.1 ± 3.4 mm, respectively; *P* < .001) and patellar tilt (26.4° ± 12.5° vs 13.2° ± 6.7°, respectively; *P* < .001) compared with patients in group I. Both groups showed similar preoperative cartilage integrity in the PF joint (not significant). Postoperatively, both groups had similar patellar chondral damage (not significant), but group II showed significantly greater trochlear chondral damage (*P* = .001 for medial; *P* < .001 for central; and *P* = .002 for lateral). In comparison to preoperatively, 92.9% to 97.6% of patients in group I had intact trochlear cartilage or an unchanged status of trochlear cartilage postoperatively compared with 36.4%to 63.6% of patients in group II; the incidence varied depending on the location (*P* = .001 for medial; *P* < .001 for central; and *P* = .008 for lateral). Among all PF parameters, only the postoperative sagittal tibial tubercle–trochlear groove distance was associated with the progression or new occurrence of chondral damage in the medial trochlea (*r* = 0.232; *P* = .045).

**Conclusion:**

The integrity of the PF chondral layer remained unchanged in most patients treated for patellar instability in the setting of trochlear dysplasia. Yet, significantly more patients who underwent trochleoplasty showed a decline in trochlear chondral status at short-term to midterm follow-up.

Trochlear dysplasia is frequently seen in patients with patellar instability and significantly increases the risk for recurrent patellar dislocations.^8,44^ Because of the pathological anatomy of the trochlear groove, the patella loses its static stabilizer and subluxates laterally, as it does not adequately engage with the trochlear groove during extension or early knee flexion.^32,34^ Trochlear dysplasia has also been identified as a risk factor for the development of patellofemoral (PF) chondral lesions, particularly patellar defects and osteoarthritis in general.^1,17,38^ Thus, surgical correction of the pathological anatomy of the trochlear groove is receiving increasing interest in the orthopaedic community.

Trochleoplasty has been shown to result in favorable clinical outcomes in patients treated for patellar instability as an isolated procedure as well as in conjunction with other corrective procedures such as medial patellofemoral ligament (MPFL) reconstruction and osteotomy.^2,22,41^ Trochleoplasty seeks to reconstruct the dysplastic trochlea, improving PF congruence, enhancing stability, and thus protecting the PF cartilage layer by reducing shear forces.^2,11,47^ Yet, only a few studies have investigated the influence of trochleoplasty on postoperative PF cartilage integrity. Schottle et al^35^ evaluated osteochondral biopsy specimens in 3 patients (aged 13, 17, and 21 years) at 6, 8, and 9 months after trochleoplasty. They reported that chondrocytes were predominantly viable, but chondral and subchondral changes with cell cluster formation and ingrowing lacunae in the cartilage layer were seen, both of which have been linked to the development of osteoarthritis.^25,36^ Nevertheless, they concluded that trochleoplasty is a safe procedure with a low risk of cartilage damage, as nearly 100% chondrocyte viability was detected throughout the cartilage layer.^35^ In a clinical study, Fucentese et al^12^ reported improved clinical scores in 44 knees at a median follow-up of 4 years after isolated trochleoplasty, but 37% of patients had cartilage lesions compared with the preoperative state, particularly on the lateral trochlea. In a long-term radiological study evaluating patients at a mean follow-up of 15 years (range, 12-19 years) after trochleoplasty, Rouanet and colleagues^31^ reported that 10 of 34 patients had preoperative PF osteoarthritis but none with Iwano stage >2. Yet, osteoarthritis was present in 33 of 34 patients at final follow-up, of whom 20 had Iwano stage >2. Accordingly, they concluded that while trochleoplasty restores PF stability, it does not prevent PF osteoarthritis in the long term, as cartilage deterioration is expected also in patients with unaddressed trochlear dysplasia.^31^ Other studies, however, have shown the positive effect of trochleoplasty on PF congruence, which is supposed to decrease shear forces and protect cartilage integrity.^2,47^ Still, the effect of trochleoplasty on cartilage integrity of the PF joint, particularly trochlear articular cartilage, is yet to be determined.

Thus, the primary purpose of the current study was to assess and compare postoperative cartilage integrity, evaluated on magnetic resonance imaging (MRI), in patients with trochlear dysplasia who underwent PF stabilizing surgery with or without trochleoplasty. It was hypothesized that patients who underwent thin-flap trochleoplasty would show less trochlear cartilage deterioration at midterm follow-up compared with patients with unaddressed trochlear dysplasia.

## Methods

Ethical approval was granted by the local research ethics committee (No. 2020-01052), and all included patients gave their written consent.

### Patients

A total of 472 consecutive patients with trochlear dysplasia in the setting of patellar instability underwent PF stabilizing surgery at our institution between January 2010 and December 2020. Treatment with PF stabilizing surgery, including trochleoplasty, tibial tubercle osteotomy (TTO), and MPFL reconstruction, was indicated in patients with symptomatic patellar instability who failed nonoperative management.

Patients were excluded from the current study if no preoperative or postoperative MRI scan with a minimum follow-up of 6 months was available (n = 311), they had undergone any previous cartilage surgery in the PF joint on the affected knee (n = 5), or they underwent concomitant derotational or coronal alignment osteotomy (n = 11). MRI was not routinely performed in asymptomatic patients, but postoperative MRI was indicated in patients for various reasons, such as subsequent meniscal injuries, persistent swelling, and anterior knee pain. Lastly, all patients with trochlear dysplasia types A and D were excluded (n = 70) because of selection bias, as patients with type A and only 1 patient with type D did not undergo trochleoplasty. Consequently, 75 patients with trochlear dysplasia types B and C who underwent patellar stabilizing surgery with or without concomitant trochleoplasty were included in this retrospective study. Of these, 42 patients did not undergo sulcus-deepening trochleoplasty as part of the treatment algorithm (group I), while 33 patients underwent trochleoplasty (group II). MRI was performed preoperatively and postoperatively for all patients at a minimum follow-up of 6 months.

### Clinical and Radiological Assessments

Clinical notes and operative reports were reviewed to determine patient age at the time of surgery, sex, body mass index, concomitant surgical procedures, complications, and reoperations. A musculoskeletal radiologist assessed the degree of trochlear dysplasia according to the Dejour classification,^7,8^ tibial tubercle–trochlear groove (TT-TG) distance,^13^ sagittal TT-TG distance,^20^ patellar angle,^26^ patellar tilt,^14^ lateral trochlear inclination,^4^ patellar morphology according to the Wiberg classification,^43^ and Caton-Deschamps index^5^ on both preoperative radiography and preoperative and postoperative MRI. All images were read independently, in random order and without any clinical information, on a picture archiving and communication system workstation certified for clinical use (MERLIN 7.1.22; Phoenix-PACS).

PF cartilage was evaluated according to Noyes and Stabler^27^ and Fucentese et al^12^ by dividing both the patellar and trochlear surfaces into 3 zones (medial, central, lateral) and grading cartilage from 0 to 4. Grade 0 indicated the presence of healthy cartilage characterized by consistent signal intensity, an intact cartilage surface, and an appropriate thickness. Grade 1 signified an anomaly in signal intensity within cartilage, with localized changes but no damage to the surface. For grade 2, there was evidence of superficial wear and tear, erosion, or ulceration, reaching a depth of no more than half of the total thickness of cartilage. In grade 3, the defect extended >50% but remained <100% of the cartilage layer’s thickness. Lastly, grade 4 indicated severe damage, with cartilage damage down to its full thickness.

### Surgical Technique and Postoperative Care

**Figure 1.**
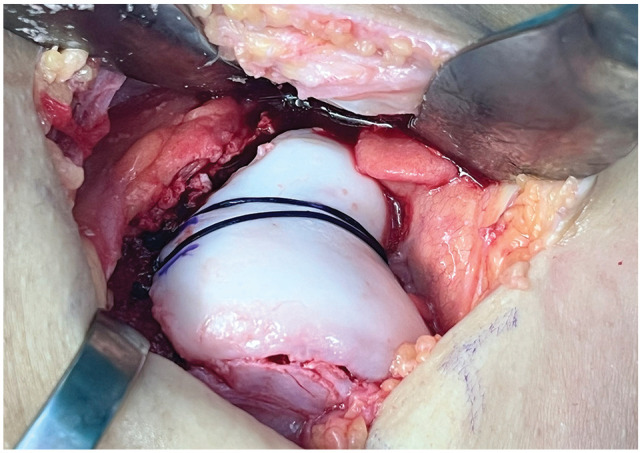
Sulcus-deepening trochleoplasty using the Bereiter technique through an open lateral approach.

All patients’ trochleae were accessed via arthrotomy through an open lateral or medial approach. Sulcus-deepening trochleoplasty was performed using the Bereiter technique.^3,12,34,42^ Accordingly, an osteochondral flake was peeled off using a curved osteotome, deepening the trochlear groove with a chisel and a high-speed bur. The osteotomy site was localized approximately 1 to 2 mm below the cartilage-bone border to achieve good plasticity of the osteochondral flake for re-creating the trochlear groove.^35^ The osteochondral flake was then fixed in the newly formed trochlea using an anchor (PopLok [ConMed] or SwiveLock [Arthrex]), loaded with 2 Vicryl No. 2 sutures (Ethicon), placed in the distal trochlear groove (Figure 1). To ensure stable fixation, the joint was brought through full range of motion. Further, all patients underwent concomitant MPFL reconstruction. Concurrent TTO was performed in patients with an increased TT-TG distance (>15 mm) or persistent lateralized patellar tracking after MPFL reconstruction and trochleoplasty when indicated. The arthrotomy site was closed using running absorbable sutures in a Z-fashion for lateral lengthening, followed by standard wound closure.

After surgery, patients were permitted protected partial weightbearing for the first 6 weeks with free range of motion. After 6 weeks, a stepwise increase to full weightbearing was allowed.

### Statistical Analysis

The sociodemographic and clinical characteristics of patients were determined using descriptive statistics. Data were assessed for normality utilizing the Shapiro-Wilk test. Subsequently, the independent *t* test or Mann-Whitney *U* test was used to compare continuous variables between the groups. Preoperative and postoperative cartilage damage were evaluated by using the chi-square or Fisher exact test. Pearson and point biserial correlation coefficients were used to assess the relationship between demographic characteristics and imaging outcomes. All statistical analyses were performed in SPSS for Mac (Version 23.0; IBM). Significance was set at *P* ≤ .05.

## Results

Patients were predominately female (68.0%) and underwent patellar stabilizing surgery at a mean age of 23.2 ± 8.0 years with a body mass index of 25.5 ± 5.0 kg/m^2^. All patients underwent MPFL reconstruction, 31 patients (41.3%) underwent concomitant TTO, 6 (8.0%) underwent microfracture, and 2 (4.8%) underwent autologous matrix-induced chondrogenesis of a focal cartilage lesion in the tibiofemoral joint.

### Clinical Outcomes

In group I, 3 patients (7.1%) sustained a patellar redislocation within the study period, while 1 patient (2.4%) complained about a feeling of instability during strenuous exercise. There were 3 patients (7.1%) who underwent revision surgery; 1 (2.4%) underwent revision MPFL reconstruction, and 2 (4.8%) underwent the removal of tibial tubercle screws after concomitant TTO.

Meanwhile, in group II, 4 patients (12.1%) sustained a patellar redislocation, while 5 patients (15.2%) complained about persistent patellar instability without a redislocation. Within the study period, 10 patients (30.3%) underwent revision surgery in group II, of which 2 cases (6.1%) were unrelated to patellar instability (anterior cruciate ligament reconstruction and meniscal repair). Revision surgery included 3 (9.1%) diagnostic arthroscopic procedures, 3 (9.1%) revision MPFL reconstructions, 3 (9.1%) subsequent TTOs, 1 microfracture (3.0%) on the patella, 1 (3.0%) lateral elevation trochleoplasty and autologous matrix-induced chondrogenesis in the lateral trochlea, and 1 (3.0%) hardware removal after concomitant TTO.

### Imaging Outcomes

Postoperative MRI was performed at a mean of 35.2 ± 26.3 months (range, 6-118 months). Overall, 45 (60.0%) and 30 (40.0%) patients had preoperative trochlear dysplasia types B and C, respectively. Patients in group II were slightly older and had a significantly higher preoperative TT-TG distance and patellar tilt compared with those in group I (*P* < .05). Postoperatively, only lateral trochlear inclination differed between the groups (*P* = .019) (Table 1).

**Table 1:** Patient Characteristics and Patellofemoral Parameters*^a^*

	Group I (n = 42)	Group II (n = 33)	*P*
Age, y	21.8 ± 8.2	25.0 ± 7.5	.032
Body mass index, kg/m^2^	25.4 ± 5.3	25.5 ± 4.7	NS
Follow-up, mo	40.0 ± 28.8	29.2 ± 21.6	NS
Trochlear dysplasia			NS
Dejour type B	24 (57.1)	21 (63.6)	
Dejour type C	18 (42.9)	12 (36.4)	
Preoperative TT-TG distance, mm	14.1 ± 3.4	18.4 ± 4.0	<.001
Postoperative TT-TG distance, mm	12.2 ± 4.6	12.2 ± 5.7	NS
Preoperative sagittal TT-TG distance, mm	2.6 ± 5.4	4.1 ± 6.3	NS
Postoperative sagittal TT-TG distance, mm	1.5 ± 4.7	2.3 ± 5.1	NS
Preoperative patellar tilt, deg	13.2 ± 6.7	26.4 ± 12.5	<.001
Postoperative patellar tilt, deg	10.6 ± 5.3	10.0 ± 6.7	NS
Preoperative lateral trochlear inclination, deg	11.8 ± 4.7	10.7 ± 4.8	NS
Postoperative lateral trochlear inclination, deg	13.2 ± 4.3	10.9 ± 3.9	.019
Preoperative Caton-Deschamps index	1.2 ± 0.2	1.2 ± 0.1	NS
Postoperative Caton-Deschamps index	1.1 ± 0.2	1.1 ± 0.1	NS
Preoperative patellar angle, deg	127.3 ± 11.5	129.7 ± 11.3	NS
Preoperative patellar morphology			NS
Wiberg type 1	2 (4.8)	2 (6.1)	
Wiberg type 2	12 (28.6)	13 (39.4)	
Wiberg type 3	28 (66.7)	18 (54.5)	

a Data are presented as mean ± SD or n (%). NS, not significant; TT-TG, tibial tubercle–trochlear groove.

Both groups showed similar preoperative cartilage integrity in the PF joint. Particularly in the trochlear cartilage layer, only 1 patient in group I and 3 patients in group II showed full-thickness cartilage damage, all in the lateral trochlea. In fact, 92.9% of patients in group I and 78.8% in group II showed pristine trochlear cartilage preoperatively. Postoperatively, both groups had similar patellar chondral integrity, but group I demonstrated a significantly better trochlear cartilage status in all 3 zones (*P* < 0.05) (Table 2).

**Table 2:** Preoperative and Postoperative Cartilage Integrity by Cartilage Status Grade*^a^*

	Group I (n = 42)					Group II (n = 33)					*P*
	0	1	2	3	4	0	1	2	3	4	
Medial patellar facet											
Preoperative	34	3	1	1	3	23	4	5	0	1	.204
Postoperative	28	2	4	3	5	18	3	4	4	4	.814
Central patella											
Preoperative	32	2	2	2	4	17	4	1	3	8	.213
Postoperative	24	3	2	5	8	10	4	5	3	11	.126
Lateral patellar facet											
Preoperative	33	5	1	2	1	21	5	2	2	3	.564
Postoperative	29	4	1	5	3	21	4	2	1	5	.440
Medial trochlea											
Preoperative	41	1	0	0	0	32	1	0	0	0	.999
Postoperative	40	2	0	0	0	19	2	2	2	8	.001
Central trochlea											
Preoperative	40	2	0	0	0	31	1	1	0	0	.493
Postoperative	38	3	0	1	0	11	4	1	2	15	<.001
Lateral trochlea											
Preoperative	39	1	1	0	1	27	3	0	0	3	.245
Postoperative	36	3	1	1	1	18	0	4	1	10	.002

a Data are presented as No.

**Figure 2.**
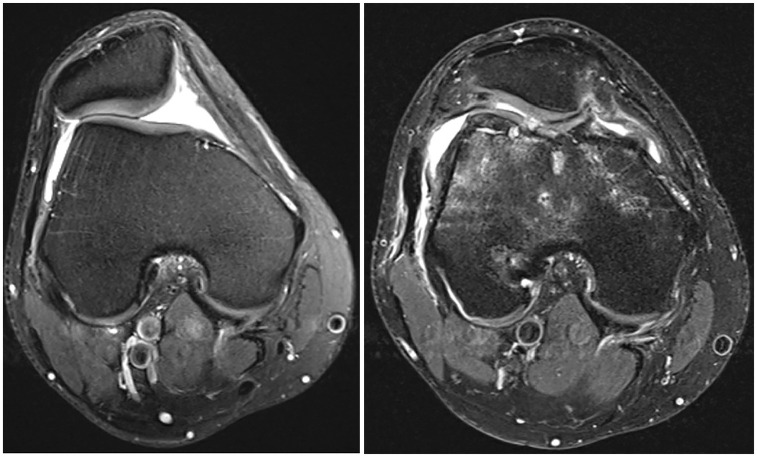
Preoperative and postoperative (at 22 months) axial magnetic resonance imaging of the patellofemoral joint in a patient who underwent medial patellofemoral ligament reconstruction, tibial tubercle osteotomy, lateral lengthening, and trochleoplasty with the progression of trochlear cartilage damage from grade 0 preoperatively to grade 4 postoperatively.

**Figure 3.**
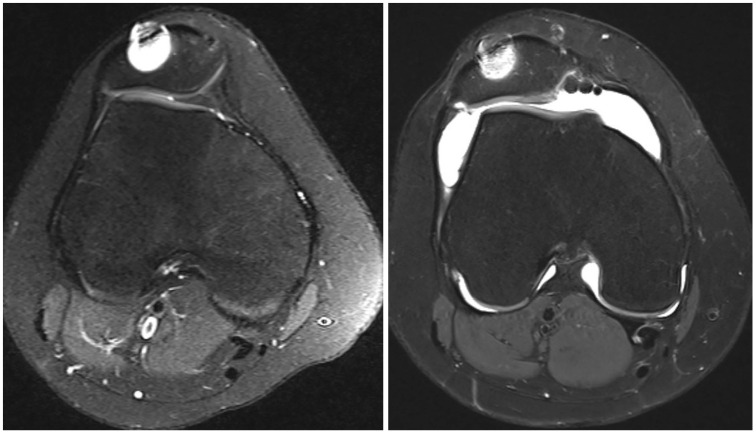
Preoperative axial magnetic resonance imaging (MRI) and postoperative magnetic resonance arthrography (at 21 months) of the patellofemoral joint in a patient who underwent revision medial patellofemoral ligament reconstruction (MRI artifact due to anchor placement in the patella) and trochleoplasty with an unchanged trochlear cartilage status of grade 0 preoperatively and postoperatively.

Compared with the preoperative state, 92.9% to 97.6% of patients in group I had intact trochlear cartilage or an unchanged status of trochlear cartilage compared with 36.4% to 63.6% in group II, depending on the location (medial, central, lateral) (*P* < .05) (Table 3 and Figures 2 and 3). Among all PF parameters, only the postoperative sagittal TT-TG distance was associated with the progression or new occurrence of chondral damage in the medial trochlea (*r* = 0.232; *P* = .045). Conversely, the progression or new occurrence of chondral damage on the lateral patellar facet was significantly correlated with a nondysmorphic patella (*r* = −0.238; *P* = .040).

**Table 3:** Cartilage Integrity Changes From Preoperatively to Postoperatively by Cartilage Status Grade*^a^*

	Group I (n = 42)					Group II (n = 33)					*P*
	0	1	2	3	4	0	1	2	3	4	
Medial patellar facet	35	0	4	1	2	22	4	4	3	0	.061
Central patella	31	5	0	3	3	24	3	3	1	2	.334
Lateral patellar facet	36	1	1	3	1	29	4	0	0	0	.161
Medial trochlea	41	1	0	0	0	20	1	2	2	8	.001
Central trochlea	40	1	0	1	0	12	3	2	2	14	<.001
Lateral trochlea	39	2	0	1	0	21	1	3	3	5	.008

a Data are presented as No.

## Discussion

The key finding of this study is that the integrity of the PF chondral layer remained unchanged in most patients treated for patellar instability in the setting of trochlear dysplasia. However, significantly more patients had an unchanged trochlear cartilage status after PF stabilizing surgery with unaddressed trochlear dysplasia.

Trochlear dysplasia is a known risk factor for patellar instability and osteoarthritis of the PF joint.^1,8,17,38,44^ Thus, corrective surgery in the form of trochleoplasty has been increasingly employed for the treatment of patellar instability. Yet, it remains unclear whether trochleoplasty is required to obtain favorable clinical outcomes in patients with patellar instability, considering the low rates of recurrent dislocations in patients with unaddressed trochlear dysplasia.^2,10,22,33,37,39,41^ Because of the structural proximity of the osteotomy site during trochleoplasty and articular cartilage, concerns regarding the development of postoperative cartilage damage have been raised.^35^ While high rates of cartilage deterioration after trochleoplasty in young patients are reported in the current literature,^12,15,31^ the present study is novel in primarily aiming to comprehensively evaluate PF articular cartilage utilizing MRI in patients with trochlear dysplasia who underwent PF stabilizing surgery with or without trochleoplasty.

Both trochlear dysplasia and sulcus-deepening trochleoplasty have been linked to the occurrence of PF cartilage lesions.^1,12,15,17,31,38^ Consequently, some authors have proclaimed that while trochleoplasty increases PF stability in patients with high-grade trochlear dysplasia, it does not prevent the development of PF osteoarthritis, which ultimately occurs in these patients with or without trochleoplasty.^31^ In a finite element analysis, Kaiser et al^18^ showed that knees with trochlear dysplasia had increased PF contact pressures through all investigated knee flexion angles (30°, 45°, 60°, and 75°) compared with healthy knees. Interestingly, simulated sulcus-deepening trochleoplasty further accentuated this difference by increasing PF contact pressures compared with the initial knees with trochlear dysplasia. The authors theorized that this may stem from the resulting smaller contact area between the often still dysplastic patella and the newly created trochlea.^18^ Conversely, Balcarek and colleagues^2^ observed that sulcus-deepening trochleoplasty improved PF congruence in patients with severe trochlear dysplasia, which likely leads to less shear forces on PF cartilage, thus protecting cartilage integrity.^47^

In fact, trochlear dysplasia has been primarily linked to cartilage lesions in the patella. Ambra et al^1^ compared 135 patients with PF cartilage lesions with 100 patients without any signs of PF cartilage damage. Of the 135 patients with PF cartilage lesions, 88 (65.2%) had patellar defects, while 47 (34.8%) had isolated trochlear cartilage damage. Their analysis revealed that trochlear dysplasia was predominantly associated with patellar lesions in patients with PF chondral defects.^1^ Similar results were reported by Holliday et al,^16^ who showed that 84.5% of patients who underwent isolated MPFL reconstruction had PF cartilage lesions preoperatively. Of those lesions, 99.1% affected the patella, whereas only 22.4% were found in the trochlea. In their patient cohort, high-grade trochlear dysplasia was strongly associated with PF cartilage lesions, showing an odds ratio of 15.7 (95% CI, 4.6-53.5; *P* < .001). Unfortunately, the authors did not distinguish further between trochlear and patellar lesions; yet, it can be assumed that primarily patellar defects were seen in patients with high-grade trochlear dysplasia, considering that most defects were seen in the patella.^16^ Interestingly, this is in accordance with the preoperative findings of the current study. Preoperatively, 47.6% of patients in group I and 57.6% of patients in group II showed patellar cartilage lesions, while only 9.2% in group I and 21.2% in group II showed any sign of trochlear cartilage damage. Postoperatively, both groups showed no significant progression of patellar cartilage lesions. For the trochlea, 92.9% to 97.6% of patients in group I had intact cartilage or an unchanged cartilage status compared with 36.4% to 63.6% of patients in group II. Hence, it can be theorized that trochleoplasty may have an influence on the integrity of trochlear cartilage in contrast to trochlear dysplasia, which seems to predominantly affect patellar cartilage.^1,16^ Yet, it must be emphasized that 60.0% of patients who underwent trochleoplasty already showed preoperative cartilage damage in the PF joint, of whom half had grade 4 cartilage defects. This may have accelerated PF cartilage deterioration, as cartilage lesions tend to progress and increase the risk for further cartilage defects.^30^

Still, the cause of cartilage lesions or the progression of osteoarthritis in the PF joint in patients with trochlear dysplasia as well as the cause of cartilage lesions/osteoarthritis in the trochlea in patients after trochleoplasty may stem from 2 distinct predispositions. It is known that unphysiological, excessive loading has a detrimental effect on articular cartilage, hence leading to cartilage deterioration and ultimately resulting in osteoarthritis of the affected joint.^23^ By reshaping the distal femur without a corresponding change to the patella, trochleoplasty can lead to altered PF congruity, decreasing the contact area with a corresponding increase in contact pressure and thus contributing to cartilage deterioration over time.^9,18^ Consequently, this would also apply to the patellar articular surface and should therefore lead to similar patellar cartilage deterioration postoperatively. Surprisingly, this has not been seen in examined cohorts. Instead, the integrity of trochlear cartilage particularly seems to be affected after trochleoplasty, even though the cartilage layer itself remains untouched during surgery. However, the subchondral bone is disturbed approximately 1.5 mm below the cartilage-bone border.^35^ The integrity of the subchondral bone is crucial for the health of overlying cartilage, with intensive biomechanical and biochemical cross-talk across this region as structures form a functional unit.^21,40,46^ Damage to the subchondral bone and subsequent remodeling can alter biological and mechanical interactions in this osteochondral unit, thus disturbing force distributions and cartilage nutrition and ultimately leading to secondary cartilage deterioration.^6,24,28,29,45,46^ Thus, it can be hypothesized that sulcus-deepening trochleoplasty may influence cartilage health by disturbing the subchondral bone, leading to biomechanical and biochemical alterations.

Further, the postoperative sagittal TT-TG distance was the only variable besides trochleoplasty that was significantly associated with postoperative trochlear cartilage lesions. This is in accordance with the current literature, as this parameter has recently been linked to PF cartilage lesions that are thought to occur because of an increase in PF loading and subsequent rise of contact stresses.^19,20^

The results of this study must be interpreted within its limitations. First, this is a retrospective analysis of patients who underwent unplanned postoperative MRI for numerous reasons (eg, persistent anterior knee pain, meniscal lesions, swelling), which may introduce selection bias. Yet, both groups were equally susceptible; hence, this should not have altered the results observed. Second, all patients were treated with the Bereiter technique using a chisel and a high-speed bur, creating a new trochlear groove close to the subchondral bone–cartilage border. The proximity to the cartilage layer may have influenced the results, and further studies need to verify the current results using other trochleoplasty techniques. Third, because of subchondral remodeling and the placement of anchors, postoperative artifacts may have been seen on MRI, potentially affecting the assessment of cartilage. However, this may only have influenced the evaluation of lesions limited to the chondral surface (cartilage status grade 1-2). Deep defects such as grades 3 and 4 were distinctly appreciated on postoperative MRI. Fourth, this study did not assess the reliability of the Dejour classification or PF cartilage grading. Yet, all MRI scans were assessed by a musculoskeletal radiologist. Fifth, the current analysis exclusively incorporated patients diagnosed with trochlear dysplasia types B and C. This selection was made because only 1 patient with trochlear dysplasia type D, who had both preoperative and postoperative MRI data, did not undergo trochleoplasty. Thus, patients with trochlear dysplasia type D were excluded from this study. Future prospective studies should also assess patients with trochlear dysplasia type D, as cartilage lesions overall may be more prominent in that patient cohort. Sixth, patients did not complete patient-reported outcome measures to objectify clinical outcomes. Still, patellar redislocations or persistent instability and reoperations within the study period were recorded and assessed for all patients included in this study. Seventh, a significant percentage of patients who underwent trochleoplasty had preoperative PF cartilage lesions, which may have negatively affected midterm PF integrity in those patients. Lastly, cartilage lesions were only graded by their depth (0-4) and not by their size. Consequently, even very small cartilage lesions had a significant effect on the observed results, while their clinical relevance may be unclear.

## Conclusion

The integrity of the PF chondral layer remained unchanged in most patients treated for patellar instability in the setting of trochlear dysplasia. Yet, significantly more patients who underwent trochleoplasty showed a decline in trochlear chondral status at midterm follow-up.
